# Surgical approach to renal tumors with cardiovascular extension in children

**DOI:** 10.1016/j.xjtc.2026.102306

**Published:** 2026-03-16

**Authors:** Valerii Iaprintsev, Amine Mazine, Michael Nightingale, Igor E. Konstantinov

**Affiliations:** a Department of Cardiothoracic Surgery, The Royal Children's Hospital, Melbourne, Australia; b Department of Paediatrics, University of Melbourne, Melbourne, Australia; c Murdoch Children's Research Institute, Melbourne, Australia; d Department of Paediatric Surgery, The Royal Children's Hospital, Melbourne, Australia; e Melbourne Children's Centre for Cardiovascular Genomics and Regenerative Medicine, Melbourne, Australia

**Keywords:** Wilms tumor, clear cell sarcoma, thrombectomy, cardiopulmonary bypass, inferior vena cava, vascular reconstruction, kidney

## Abstract

**Objectives:**

Pediatric renal tumors with intravascular extension require complex surgery. The extent of venous and intracardiac involvement determines the operative strategy. We report 3 children with unilateral renal tumors and intravascular extension who underwent complete tumor resection using tailored surgical approaches.

**Methods:**

We retrospectively reviewed 3 pediatric cases of renal tumors with intravascular extension. Accompanying surgical videos demonstrate the operative techniques.

**Results:**

All patients had primary renal tumors extending into the ipsilateral renal vein and inferior vena cava, with variable extension into the right atrium. Two children had Wilms tumor with partial response to neoadjuvant chemotherapy. The third had clear cell sarcoma of the kidney that did not respond to therapy and required urgent surgery for progressive heart failure and risk of tricuspid valve obstruction. Surgical strategies were individualized according to the level of vascular involvement. Complete resection with negative margins and inferior vena cava preservation was achieved in all patients. All children were successfully extubated within 2 to 5 days and discharged within 2 weeks postsurgery.

**Conclusions:**

Complete en bloc resection of pediatric renal tumors with intravascular extension is feasible and safe with meticulous planning and appropriate use of cardiopulmonary bypass. Preservation of venous return and contralateral renal function is essential. The accompanying videos illustrate key technical steps to achieve safe and effective resection.

**Figure:**
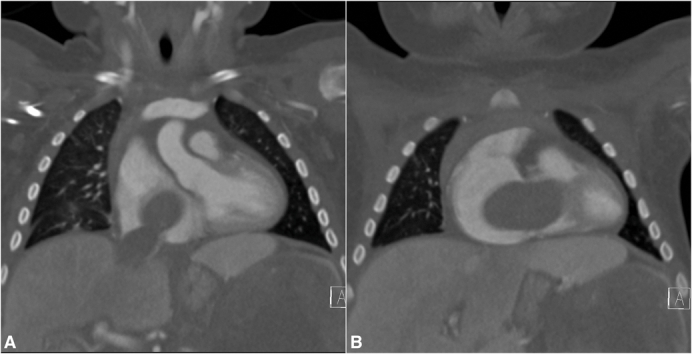
Preoperative CT of a patient with Wilms tumor with cardiovascular extension.

**Central Message**

Complete resection of renal tumors with vascular extension can be achieved safely while preserving contralateral renal function and avoiding resection of the inferior vena cava.

**Perspective**

Children with renal tumors and vascular extension face high surgical risk. A coordinated, multidisciplinary approach enables complete tumor resection while maintaining venous integrity and protecting the function of the remaining kidney.

Kidney tumors are the most common retroperitoneal neoplasms associated with vascular extension.^1^ Wilms tumor is the predominant type, with an incidence of about 18.4 cases per 1,000,000 children younger than 4 years of age and vascular invasion in 4 to 10% of cases.^2^, ^3^, ^4^ Although overall outcomes are favorable, complete resection of the primary tumor and its intravascular extension remains essential. Total excision improves survival to rates comparable with those without vascular involvement, exceeding 85% at 5 years.^5^^,^^6^ This underscores the need for surgical strategies that enable safe and complete tumor removal.

The extent of venous thrombus is the main determinant of operative approach. Daum and colleagues^7^ classified thrombus extension into 4 levels: infrahepatic, retrohepatic, suprahepatic, and atrial; some authors include a fifth level when the thrombus extends into the right ventricle. Depending on the level, cardiopulmonary bypass (CPB) or venovenous bypass may be required. In addition to nephrectomy, resection may include cavotomy or cavectomy with or without inferior vena cava reconstruction.^8^^,^^9^

In unilateral tumors, preservation of the remaining kidney is critical. Occlusion of the contralateral renal vein in the absence of collateral drainage can cause venous congestion and ischemic injury of the unaffected kidney.^10^ To mitigate this risk, some authors recommend establishing a temporary venous shunt between the renal and portal veins during inferior vena cava (IVC) resection.^11^ Here, we describe the surgical techniques used at our institution to achieve complete en bloc resection of renal tumors with intravascular extension while preserving contralateral renal function. The study was approved by the Royal Children's Hospital Human Research Ethics Committee HREC/21/QCHQ/80891 on November 11, 2021, and the parents of the children provided informed written consent for inclusion in this report.

## Patient 1

A 3-year-old boy with Beckwith-Wiedemann syndrome presented with a palpable left abdominal mass. Ultrasound and computed tomography (CT) of the abdomen revealed a large left renal tumor invading the left renal vein, IVC, and right renal vein, as well as a solitary 10-mm lingular pulmonary metastasis. Biopsy confirmed stage IV Wilms tumor. After 6 weeks of neoadjuvant chemotherapy, follow-up CT demonstrated approximately 50% reduction in tumor volume from 865 mL to 450 mL and partial regression of the IVC thrombus, which remained 5 cm below the right atrium. No new pulmonary or solid-organ metastases were identified.

**Figure 1:**
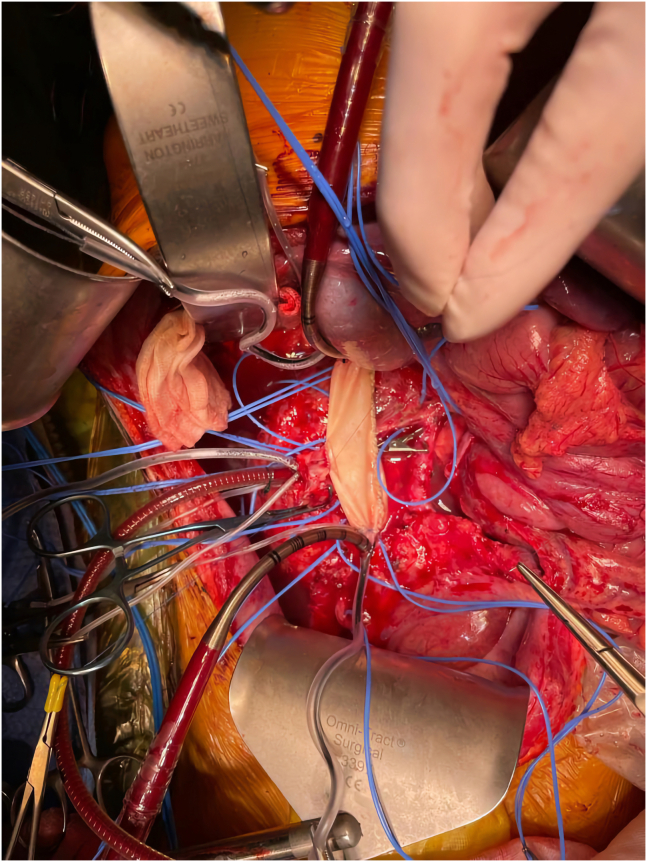
Intraoperative photograph showing the venous cannulation sites and inferior vena cava reconstruction using a bovine pericardial patch in patient 1.

A left radical nephrectomy with en bloc resection of the IVC and a segment of the right renal vein was performed under venovenous bypass. Through a bilateral subcostal laparotomy incision, the tumor-bearing kidney and involved venous segments were mobilized and the left renal artery ligated. Bypass was established between the infrahepatic IVC, the infrarenal IVC and right renal vein to maintain unobstructed venous drainage during procedure (Figure 1). The right renal vein cannula was inserted through a small venotomy on the anterolateral wall of the vein near its hilum and was kept in situ during the procedure. The IVC was opened, and the adherent thrombus and kidney were excised en bloc. The cardiac team reconstructed the IVC with a bovine pericardial patch and reimplanted the right renal and gonadal veins. Bypass time was 101 minutes.

The patient was extubated on postoperative day 1 and transferred to the ward on day 2. He was discharged home 2 weeks after surgery. One week later, he underwent thoracoscopic resection of the residual pulmonary metastasis, followed by completion of adjuvant chemotherapy 1 year postoperatively. Two years after the initial operation, he developed a solitary hepatic recurrence (segment II, 4 × 5 cm) that was successfully resected via wedge excision. The postoperative course was uneventful, and relapse treatment was completed according to the International Society of Paediatric Oncology Umbrella protocol. The patient remains alive and disease-free 2 years after the index surgery.

## Patient 2

**Figure 2:**
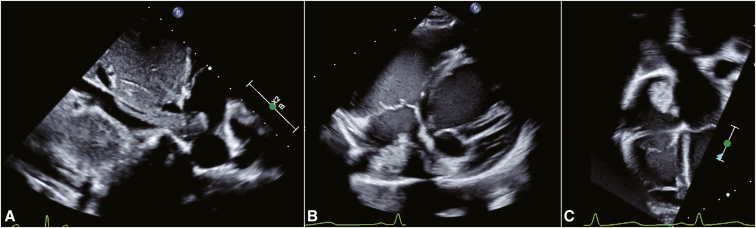
Preoperative (A) subcostal and (B and C) transthoracic echocardiography of patient 2 showing the intracardiac component of the tumor thrombus.

**Figure 3:**
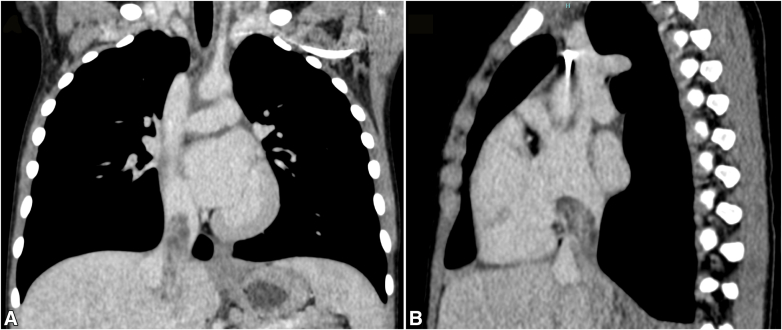
Preoperative contrast-enhanced computed tomography of patient 2. Coronal (A) and sagittal (B) images demonstrating intravascular tumor thrombus extending into the inferior vena cava and right atrium.

A 4-year-old boy with a triphasic left Wilms tumor (grade III on biopsy, no anaplasia) was found to have tumor thrombus extending from the left renal vein into the IVC, right atrium (RA), and across the tricuspid valve on echocardiography and computed tomography (Figures 2 and 3). He was transferred to our institution for definitive surgical management. After 4 cycles of neoadjuvant chemotherapy, the renal mass decreased in size; however, the intracardiac thrombus persisted with continued tricuspid valve contact. Cytogenetic analysis showed chromosome 1q gain, a finding associated with greater recurrence risk and reduced long-term survival in Wilms tumor.^12^

A left nephrectomy with en bloc excision of the intracaval and intracardiac tumor was performed (Video 1). The pediatric surgical team undertook a subcostal muscle-cutting laparotomy, followed by a median sternotomy for cardiac access, with radial division of the diaphragm toward the cavoatrial junction. After systemic heparinization, CPB was established via cannulation of the ascending aorta, superior vena cava, and infrarenal IVC. After aortic crossclamping and administration of cardioplegia, a right atriotomy extended inferiorly into the IVC to the infrarenal level revealed a confluent tumor thrombus extending from the left renal vein through the IVC into the RA. The retrohepatic cavotomy was placed on the right lateral wall to preserve hepatic venous inflow and facilitate patch closure. Hepatic venous anatomy was directly visualized. The incision allowed mobilization of the thrombus from the vessel wall and complete en bloc resection of the tumor together with its thrombotic extension. Macroscopic caval wall invasion was defined by fixed adherence with absence of a safe dissection plane and/or gross wall infiltration requiring full- or partial-thickness excision. A fibrous reaction was suspected when a clear plane could be developed and the caval wall appeared intact after tumor mobilization. Short periods of Pringle maneuver—ie, temporary clamping the blood vessels in the hepatoduodenal ligament to control hepatic blood inflow—facilitated safe removal. The IVC-RA junction was reconstructed primarily, and the remaining IVC was augmented with a bovine pericardial patch from the infrarenal segment to the diaphragm. The diaphragm was reconstructed with continuous PDS suture at the end of the procedure. No clinically significant diaphragmatic dysfunction was observed at follow-up. CPB time was 76 minutes and aortic crossclamp time was 24 minutes.

Histopathology confirmed mixed-subtype Wilms tumor (60% epithelial, 40% stromal) with nonviable tumor present in 1 of 9 lymph nodes (stage III, nodal involvement). Postoperative echocardiography demonstrated a patent IVC-right atrium junction, no residual thrombus, trivial tricuspid regurgitation, and preserved right ventricular function. The patient was extubated on postoperative day 2, transferred from intensive care unit on day 4, and returned to the referring hospital for continuation of adjuvant chemoradiotherapy. At 2-month follow-up, the child remains well and disease-free.

## Patient 3

**Figure 4:**
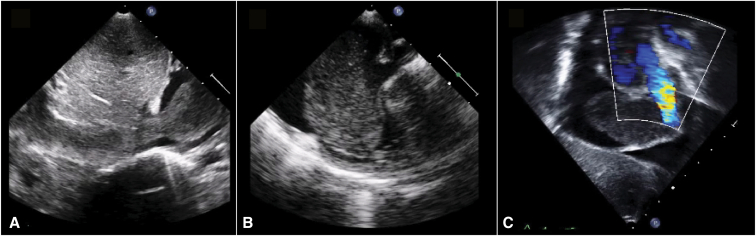
Preoperative (A) subcostal and (B and C) transthoracic echocardiography of patient 3 showing the intracardiac component of the tumor thrombus.

**Figure 5:**
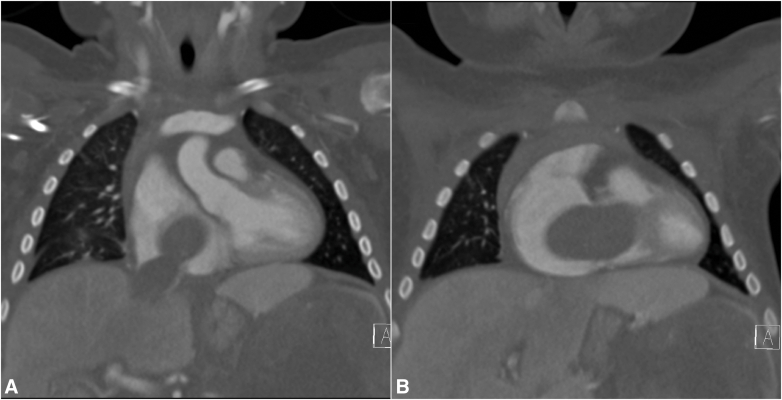
Preoperative computed tomography of patient 3. Coronal contrast-enhanced computed tomography images showing a large left renal mass with intravascular extension (A) reaching the right atrium and (B) right ventricle.

A 1-year-old boy with stage III clear cell sarcoma of the left kidney presented with progressive edema, ascites, and fatigue. Echocardiography (Figure 4) and CT (Figure 5) showed confluent tumor extension from the left renal vein into the IVC, right atrium, and right ventricle, causing severe tricuspid obstruction. Within 2 days, his condition deteriorated (central venous pressure increased from 13 to 18 mm Hg, mixed venous oxygen saturation 53%, hypotension, bradycardia). Because of the high risk of complete right ventricular inflow obstruction, urgent surgery was undertaken.

Bilateral subcostal laparotomy and median sternotomy were performed for optimal exposure (Video 2). The pediatric surgical team first carried out a left nephrectomy with en bloc tumor excision. Left visceral rotation, ureteral division, and isolation of the renal vessels enabled intact removal of the kidney and retroperitoneal mass. CPB was then established via the aorta, superior vena cava, infrarenal IVC, and right renal vein. After cardioplegic arrest, right atriotomy revealed tumor that was not adherent to the endocardium or tricuspid valve. The longitudinal venotomy in the retrohepatic IVC segment was created along the right lateral wall, extending caudally from the cavoatrial junction, to facilitate safe exposure and patch reconstruction while preserving hepatic venous inflow. The intracardiac and caval components were removed en bloc through the atriotomy and venotomy. The patent foramen ovale and atriotomy were closed, and the IVC and right renal vein were reconstructed with a bovine pericardial patch. CPB time was 88 minutes and aortic crossclamp time was 10 minutes.

The patient was extubated on postoperative day 3 and discharged from the intensive care unit on day 5. He received adjuvant radiotherapy 1 month after surgery and the chemotherapy course was subsequently completed. At 2-year follow-up, the child remains well and disease-free, with preserved right renal function.

In all patients, anticoagulation was commenced postoperatively after hemostasis was secured, using an unfractionated heparin infusion (10-15 units/kg/h) with dose adjustment to achieve an aPTT of ∼60 to 80 seconds. This was continued for 10 days, followed by 6 months of aspirin.

## Discussion

We report on 3 pediatric patients with complex unilateral renal tumors with intravascular extension (Table 1). Two children had Wilms tumor with variable venous involvement; the third had clear cell sarcoma of the kidney. In all cases, the affected kidney and associated intravascular component were excised en bloc, irrespective of the thrombus extent. Neoadjuvant chemotherapy was administered according to the International Society of Paediatric Oncology Wilms tumor protocol (vincristine, actinomycin D, and doxorubicin) in all patients, including the third child in whom Wilms tumor was initially presumed.^13^^,^^14^ Chemotherapy substantially reduced the primary tumor volume in patients 1 and 2 and resulted in mild thrombus regression; it had no appreciable effect in patient 3.

**Table 1:** Characteristics and outcomes of pediatric patients undergoing resection of renal tumors with venous extension

Variable	Patient 1	Patient 2	Patient 3
Age, y	3	4	1
Sex	Male	Male	Male
Diagnosis	Wilms tumor	Wilms tumor	Clear cell sarcoma
International Society of Paediatric Oncology stage	II (clinically IV)	III	III
Genetic findings	–	1q gain	–
Laterality	Left	Left	Left
Extent of vascular invasion	IVC, RRV	IVC, RA	IVC, RRV, RA, RV
Tumor level	III	IV	IV
Preoperative chemotherapy	Yes (6 cycles)	Yes (4 cycles)	Yes (1 cycle VAD)
Response to chemotherapy	Partial	Partial	Minimal
Bypass strategy	Veno-venous	CPB	CPB
Cannulation sites	IVC (suprahepatic), RRV, IVC (infrarenal)	Aorta, SVC, infrarenal IVC	Aorta, SVC, RRV, infrarenal IVC
Bypass time, min	101	76	88
Crossclamp time, min	–	24	10
IVC reconstruction	Bovine pericardial patch	Bovine pericardial patch	Bovine pericardial patch
Intraoperative complications	None	None	None
Postoperative complications	None	None	Ileus
ICU duration, d	2	4	5
Hospital duration, d	14	15	11
Adjuvant therapy	Completed	Completed	Completed
Remaining kidney function	Not affected	Not affected	Not affected
Follow-up duration	2 y	2 mo	1.5 y
Creatinine, μmol/L∗	33	35	16
Urea, mmol/L∗	5.2	6.3	1.3
Current status	Alive, disease-free	Alive, disease-free	Alive, disease-free

*IVC*, Inferior vena cava; *RRV*, right renal vein; *RA*, right atrium; *RV*, right ventricle; *CPB*, cardiopulmonary bypass; *SVC*, superior vena cava; *ICU*, intensive care unit.

∗ Measurements at last follow-up.

Because intravascular tumor extension persisted despite chemotherapy, CPB or venovenous bypass was required in all cases. Venovenous bypass was used in patient 1; full CPB was necessary in patients 2 and 3 because of intracardiac thrombus. Despite its risks, CPB offers important advantages. It provides excellent exposure, facilitates complete en bloc excision, and reduces the risk of thrombus fragmentation and embolization. This is critical, as thrombus division constitutes tumor spill in Children's Oncology Group/National Wilms Tumor Study Group protocols, upstages the patient to stage III, and contributes to decreased long-term survival and markedly increased recurrence risk.^5^ CPB also increases the likelihood of preserving the native IVC and facilitates its reconstruction in most cases. When feasible, we preserve as much native IVC tissue as possible following tumor and thrombus removal and reconstruct the resected segment with an acellular bovine pericardial patch. In the described patients, IVC wall resection was reserved for segments demonstrating macroscopic invasion (fixed adherence and absent safe dissection plane). Intracardiac extension alone was not interpreted as transmural invasion; decisions regarding wall resection were based on intraoperative assessment of the caval wall/tumor interface. Alternatively, replacement of the resected IVC segment with a prosthetic graft has also been described,^15^ but outcomes are poor because of graft thrombosis in nearly all cases.^16^

Preservation of venous return is equally important to protect the contralateral kidney. Occlusion of the contralateral renal vein in the absence of collateral drainage may result in venous congestion, impaired perfusion, and ischemic injury.^10^ Temporary renal-portal venous shunting during IVC resection has been proposed to mitigate this risk.^11^ Crossclamping of the IVC may also reduce venous return, even in patients with chronic IVC occlusion,^17^ and can produce hemodynamic instability. For these reasons, we consider maintenance of full venous return during reconstruction essential. CPB was conducted under mild hypothermia (32 to 34 °C) at full calculated flow for body surface area. The Pringle maneuver was applied intermittently, with individual episodes of inflow occlusion kept to <5 minutes. Hypothermic circulatory arrest was considered but not required, given adequate venous decompression and clamp control. In addition, adequacy of renal venous drainage was monitored intraoperatively by kidney appearance (turgor, color, capsular congestion), stability of venous return and line pressures, reservoir filling trends, and urine output. Cannula position and size were adjusted if any congestion or impaired drainage was suspected. We favored central venous cannulation over femoral cannulation because direct central access was technically straightforward, ensured reliable drainage, and allowed coordinated abdominal and cardiac exposure, without the need for groin exposure.

Previous reports have suggested that thrombotic IVC obstruction is often associated with firm endothelial adhesion, complicating thrombectomy.^9^ However, our experience indicates that en bloc thrombus removal with limited IVC wall resection is feasible in most cases, even with near-complete occlusion. Similar observations have been reported in adults with renal cell carcinoma, where tumor thrombi can often be mobilized from the vessel wall with relative ease. Routine venous reconstruction appears important to prevent postoperative venous insufficiency in these patients.^17^^,^^18^

In addition, urgent surgery may be required when thrombus progresses despite chemotherapy or when cardiac function becomes compromised. Indications include tumor rupture, heart failure symptoms, high embolic risk, or extension into the right ventricle.^2^^,^^19^ In our series, patient 3 required emergency surgery because of progressive heart failure and impending tricuspid obstruction. Even in this urgent setting, the operative strategy described in this article provided safe exposure and allowed controlled, complete excision.

## Conclusions

Complete en bloc resection of pediatric renal tumors with intravascular extension can be performed safely with meticulous planning and appropriate use of CPB. Preservation of venous return and contralateral renal function is paramount. Our experience supports the feasibility of IVC reconstruction, even in extensive thrombus involvement. The accompanying videos illustrate key operative steps to guide surgical strategy in these complex cases.

## Conflict of Interest Statement

The authors reported no conflicts of interest.

The *Journal* policy requires editors and reviewers to disclose conflicts of interest and to decline handling or reviewing manuscripts for which they may have a conflict of interest. The editors and reviewers of this article have no conflicts of interest.

## Supplementary Data

Video 1 Surgical technique used for tumor removal and vascular reconstruction in patient 2. Video available at: https://www.jtcvs.org/article/S2666-2507(26)00113-6/fulltext.

Video 2 Surgical technique used for tumor removal and vascular reconstruction in patient 3. Video available at: https://www.jtcvs.org/article/S2666-2507(26)00113-6/fulltext.
