# Supplementary figures and images for: Surgical approach to renal tumors with cardiovascular extension in children
Source: JTCVS Tech. 2026 Mar 16;37:102306. doi: 10.1016/j.xjtc.2026.102306 (PMC13261278; doi:10.1016/j.xjtc.2026.102306)

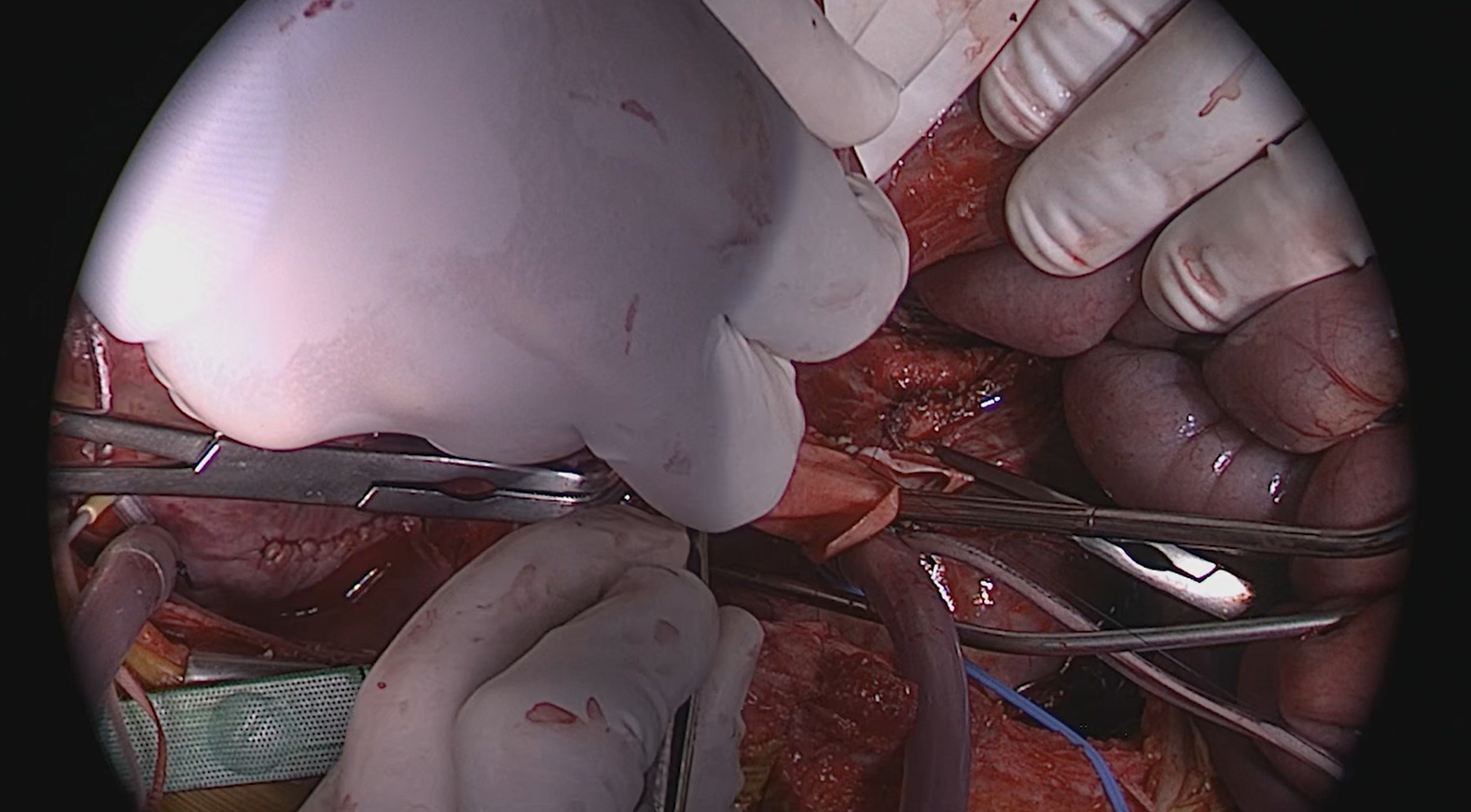

Supplement: Video 1 — Surgical technique used for tumor removal and vascular reconstruction in patient 2. Video available at: https://www.jtcvs.org/article/S2666-2507(26)00113-6/fulltext. [file fx2.jpg]

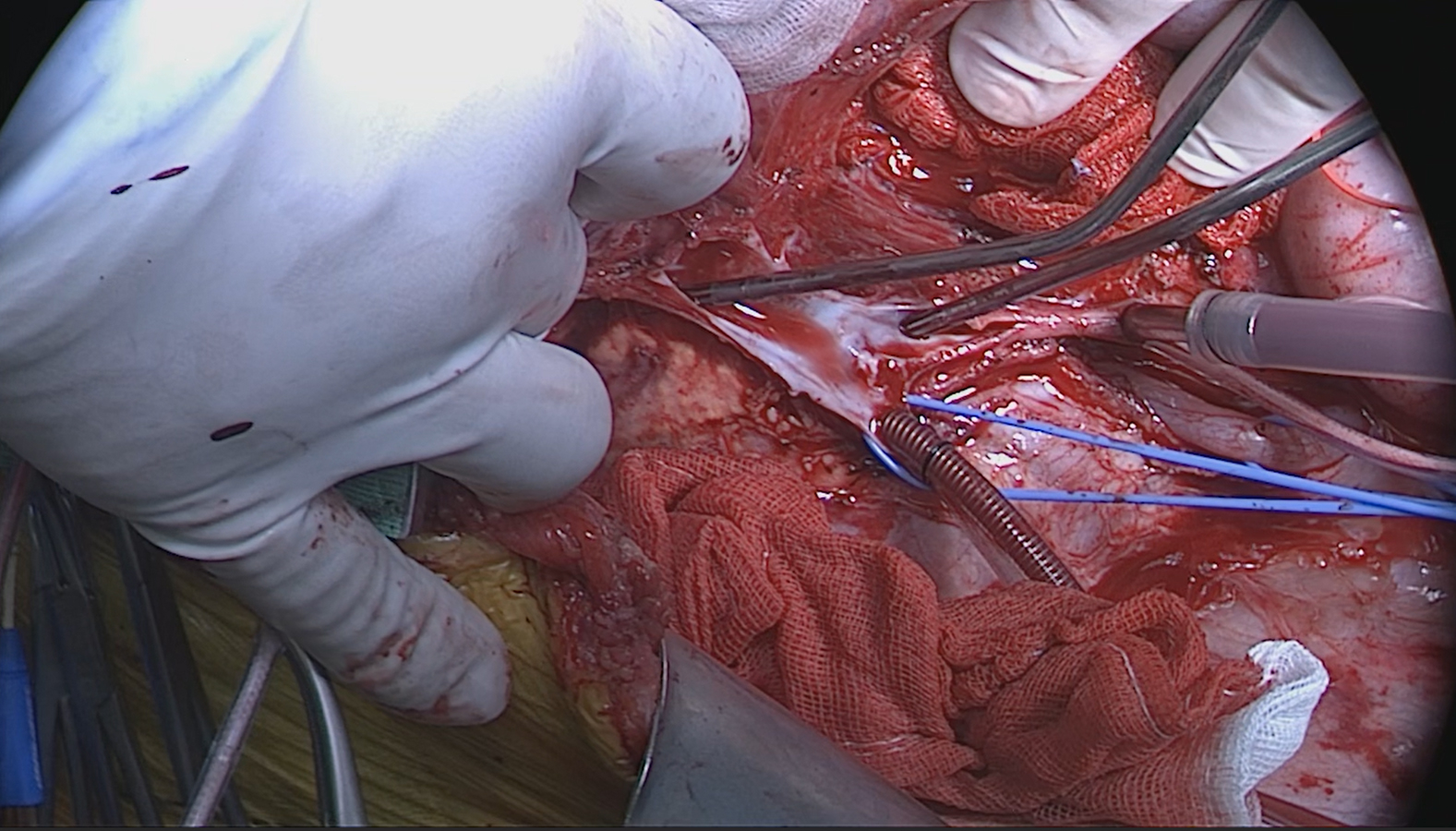

Supplement: Video 2 — Surgical technique used for tumor removal and vascular reconstruction in patient 3. Video available at: https://www.jtcvs.org/article/S2666-2507(26)00113-6/fulltext. [file fx3.jpg]
